# Cancer-associated thrombosis in Asia

**DOI:** 10.1186/s12959-016-0110-4

**Published:** 2016-10-04

**Authors:** Pantep Angchaisuksiri

**Affiliations:** Division of Hematology, Department of Medicine, Faculty of Medicine Ramathibodi Hospital, Mahidol University, 270 RAMA VI Road. Rachathevi, Bangkok, 10400 Thailand

**Keywords:** Cancer, Thrombosis, Asian

## Abstract

Thrombosis is a common complication in cancer patients. Although the major inherited risk factors for thrombophilia are different between Asians and Caucasians, the main acquired risk factors that are associated with the development of venous thromboembolism (VTE) in Asians appear to be similar to those for Caucasians. Malignancy is the most important acquired risk factor for VTE in Asians. Recent studies have shown that the incidence of VTE is significant in Asian patients with cancer, particularly those in an advanced stage. Cancer associated VTE is more severe and associated with higher morbidity and mortality. Unprovoked VTE is associated with an increased risk of subsequent cancer diagnosis. A number of international and national guidelines are available to provide guidance to healthcare providers to treat and prevent this serious complication based on best-available evidence. Identifying cancer patients at risk for VTE and use of appropriate thromboprophylaxis in these patients can potentially improve morbidity and mortality. Although direct oral anticoagulants (DOACs) may become an attractive treatment for cancer-associated VTE, further clinical trials are needed to evaluate the safety and efficacy of DOACs directly against LMWH in cancer patients.

## Background

Although the major inherited risk factors for thrombophilia are different between Asians and Caucasians, the main acquired risk factors that are associated with the development of venous thromboembolism (VTE) in Asians appear to be similar to those for Caucasians. Malignancy is the most important acquired risk factor for VTE in Asians. About 16–40 % of VTE cases are cancer-associated [1].

## Review

### Cancer-associated thrombosis in Asia

Several recent studies have reported the incidence of VTE among cancer patients in Asia. A Korean population-based study analyzed VTE occurrences after curative cancer surgery between 2007 and 2011 [2]. The incidence of symptomatic VTE after curative surgery in stomach cancer was 0.47 % among 87,409 patients. Independent risk factors identified for VTE were older age, female sex, and receiving general anesthesia. In patients with metastatic stomach cancer, the two-year cumulative incidence of all VTE increased to 24.4 % [3]. Risk factors for developing VTE were advanced stage, older age, and no major surgery. Another study of symptomatic VTE cases showed 3.5 % one-year cumulative incidence of VTE in patients with inoperable advanced stomach cancer [4]. The incidence of VTE after colorectal cancer surgery was higher than that for stomach cancer. The Korean nationwide study reported a 1.67 % incidence of symptomatic VTE among 73,961 patients [2] and the single-center study showed a similar incidence approaching 1 % [5]. The incidence of all VTE events in patients with metastatic colorectal cancer was 12.6 %, including incidentally detected VTE [6]. The incidence of VTE in Korean patients with loco-regional colorectal cancer was lower than in Western patients. However, the incidence of VTE in Korean patients with advanced cancer was not lower than in Western patients. It implies that the protective effect of Asian ethnicity on VTE development disappears as tumor stage increases [6].

Studies involving other types of cancers in Asia have also been published. A Taiwanese population-based study of 43,855 newly diagnosed cancer patients was recently reported [7]. The VTE incidence rates were 9.9 per 1000 person-years. Certain cancers, such as liver, pancreas, and lung, have higher incidence rates. Previous VTE, cancer site, chemotherapy, and major surgery were significant VTE risk factors. In Korean patients with pancreatic cancer, the incidence of VTE was 5.3 % [8] as compared with 10–20 % reported in Western countries [9, 10].

The most common cancer associated VTE in Thailand were gynecologic cancers, followed by gastrointestinal and hepatobiliary cancers, lung cancer, and lymphoma [11, 12]. Postoperative DVT occurred more frequently in cancer patients than in non-cancer patients (21.1 vs 11.9 %). Cancer associated VTE was found to be more severe and associated with higher morbidity and mortality [12].

Another Taiwanese population-based study investigating the relationship between unprovoked VTE and cancer risk showed that the risk of cancer was significantly higher in the unprovoked VTE patients (hazard ratio 2.3). The risk was increased in the first 6 months after VTE. Therefore VTE can be a presenting symptom of occult cancer [13]. Furthermore, the mortality risk of cancer patients with unprovoked VTE was significantly higher than that for cancer patients without VTE at one year.

An analysis of VTE in 497,180 Taiwanese cancer patients with the development and validation of a risk-stratification scoring system was recently published [14]. It was found that patients with a previous history of VTE and female ages between 40 and 80 years old had high risk of VTE. Patients with myeloma, prostate cancer, lung cancer, gynecologic cancers, sarcoma, and metastasis of unknown origin had relatively high rate of VTE. A risk-stratification scoring system was developed to classify patients with cancer into four risk categories (very low risk, low risk, intermediate, and high risk). VTE incidence in each category was 0.5, 0.9, 1.5, and 8.7 %, respectively. The risk scoring system could be helpful in decision making concerning thromboprophylaxis in patients with cancer.

### Management of cancer-associated thrombosis

Several international and national organizations have published clinical practice guidelines. The most widely used guidelines include those developed by the American College of Chest Physicians (ACCP) [15] and the American Society of Clinical Oncology [16]. All major international guidelines agree on the following statements:

- Initial treatment of VTE – Low molecular weight heparin (LMWH) is preferred over unfractionated heparin except in patients with severe renal impairment.
- Long-term treatment of VTE – LMWH is preferred over vitamin K antagonist (VKA) therapy except in patients with severe renal impairment.
- Treatment of catheter-related thrombosis – Anticoagulation should be used as in treatment of VTE.
- Prevention of VTE in ambulatory patients receiving chemotherapy –Anticoagulant prophylaxis is not recommended except in patients receiving thalidomide- or lenalidomide-based regimens for multiple myeloma.
- Prevention of VTE in hospitalized medical patients – Anticoagulant prophylaxis should be given to all cancer patients admitted with an acute medical illness who do not have a contraindication.
- Prevention of VTE in surgical patients – Anticoagulant prophylaxis should be given to cancer patients undergoing major surgery. Extended prophylaxis up to 1 month should be considered for those undergoing major abdominal or pelvic surgery.
- Prevention of catheter-related thrombosis – Anticoagulant prophylaxis is not recommended.

National guidelines on VTE prevention have recently been published in some Asian countries to encourage thromboprophylaxis in Asia. The original Korean guideline on VTE prevention was a consensus of opinions of an expert panel [17]. The guideline was updated in 2014 based on the 9th ACCP guidelines and assigned risk stratification based on the results of a study on the incidence of VTE after major surgery [18]. The guideline also used the quality of evidence and strength of recommendations from the 9th ACCP guidelines.

Recently, the Asian Venous Thromboembolism Guideline has updated its recommendations for VTE prevention [19]. This guideline represents the views of the Asian Venous Thrombosis Forum, which comprised participants from China, Hong Kong, India, Korea, Malaysia, Philippines, Singapore, Taiwan, and Thailand. Available data on VTE from the Asian region were reviewed and recommendations were tailored to the needs of the region. The working group makes the following recommendations:

- LMWH should be considered in surgery for cancers with high risk of VTE. Mechanical prevention using intermittent pneumatic compression or graduated compression stockings should be considered instead of LMWH in patients with high bleeding risk.
- In patients undergoing chemotherapy, adequate hydration and frequent mobilization decreases the risk of VTE. Pharmacological prophylaxis may not be indicated unless there are other risk factors.

### The use of direct oral anticoagulants in cancer-associated thrombosis

The development of direct oral anticoagulants (DOACs) that directly inhibit factor Xa (rivaroxaban, apixaban and edoxaban) or thrombin (dabigatran) is an advance in the prevention and treatment of VTE. These agents are more convenient as they are taken by mouth in fixed once or twice daily doses, have few drug and food interactions and do not require laboratory monitoring. These drugs have been demonstrated to be as effective as conventional therapy with warfarin for acute VTE treatment and have been approved for VTE treatment. However, DOACs were not studied in cancer-specific populations. The results of this patient subgroup have recently been published [20–23]. A systematic review and meta-analysis of the efficacy and safety of DOACs in patients with VTE and cancer suggested that DOACs may be as effective and safe as conventional treatment [24].

Despite the undeniable practical advantages of DOACs over VKA and LMWH for the prevention and treatment of cancer-associated thrombosis, there are important and clinically relevant concerns regarding the extrapolation of published results to the cancer population. These include the small number of highly selected cancer patients (2.6–9.3 %; average 5.8 %) enrolled in each study [25–30] and the use of warfarin or placebo rather than LMWH in the control arm. In addition, although these anticoagulants have fewer drug interactions than VKAs, interactions do exist with some chemotherapeutic agents. Cyclosporine, tacrolimus, tamoxifen, lapatinib, nilotinib, and sunitinib increase DOACs plasma levels, whereas dexamethasone, doxorubicin, and vinblastine reduce DOACs plasma levels [31]. Whether these interactions are clinically significant is not known. Finally, gastrointestinal problems in patients with cancer can potentially alter drug delivery and absorption.

Before DOACs become an accepted treatment option for cancer-associated VTE, they have to be evaluated in a head-to-head comparison with LMWH. Clinical studies are needed to answer this question and the many other unmet clinical needs in patients with cancer-associated thrombosis. Studies designed to evaluate the efficacy and safety of DOACs in cancer-associated VTE compared to LMWH have been initiated [32].

## Conclusion

Although in general, Asian patients have a lower incidence of VTE than Caucasian patients, VTE incidence is significant in Asian patients with cancer, particularly those in an advanced stage. A number of guidelines are available to provide guidance to healthcare providers to treat and prevent this serious complication. Identifying patients most at risk for VTE and use of appropriate thromboprophylaxis in these patients can potentially improve morbidity and mortality. Although DOACs may become an attractive treatment for cancer-associated VTE, further clinical trials are needed to evaluate the safety and efficacy of DOACs directly against LMWH in cancer patients.
